# Stimulation selectivity in transcranial motor evoked potentials for monitoring during surgery for supratentorial lesions

**DOI:** 10.1016/j.cnp.2026.01.006

**Published:** 2026-01-30

**Authors:** Tammam Abboud, Jan-Bernd Wemhoff, Fares Komboz, Angelina Nazarenus, Tatiana Chacon, Dorothee Mielke, Veit Rohde

**Affiliations:** a Dept. of Neurosurgery, University Medical Center Göttingen, Germany; b Dept. of Neurosurgery, University Medical Center Augsburg, Germany

**Keywords:** Motor threshold, False positive, Intraoperative monitoring, Motor evoked potentials, Brainstem

## Abstract

**Objective:**

To identify the most selective electrode configuration for monitoring of transcranial motor evoked potentials (TcMEP) during supratentorial tumor surgery and determine the stimulation threshold at which bilateral corticospinal tract (CST) activation occurs at deeper white matter levels.

**Methods:**

Fifty-six patients undergoing resection of supratentorial tumors without preoperative motor deficits were prospectively included. TcMEPs were elicited under general anesthesia using three electrode configurations: C1 ↔ C2, C3 ↔ C4, and C3/4 → Cz. Motor thresholds (MT) were recorded bilaterally from the abductor pollicis brevis (APB), and the selectivity ratio (ipsilateral/contralateral MT) was calculated. Electrode combinations were compared using paired t-tests. Linear regression assessed the influence of age, sex, height, and weight.

**Results:**

The C3/4 → Cz montage demonstrated the highest selectivity ratio (median 248%, range: 140–364%), significantly greater than C1 ↔ C2 and C3 ↔ C4 (p < 0.001). Bilateral CST activation with C3/4 → Cz began at 140% of the contralateral MT. Age correlated with lower MTs; sex, height, and weight had no significant effect.

**Conclusions:**

C3/4 → Cz is the most selective configuration for eliciting contralateral TcMEP and requires higher stimulation intensity before activating bilateral CST fibers at deeper levels.

**Significance:**

This study supports using C3/4 → Cz to optimize TcMEP selectivity and reduce the risk of false-negative monitoring due to deep white matter stimulation.

## Highlights

• Selectivity was compared across three transcranial motor evoked potential setups.
• C3/4 → Cz electrode montage yielded the highest selectivity ratio.
• Age influenced stimulation thresholds; height, weight, and sex did not.

## 1 Introduction

Intraoperative neuromonitoring (IONM) of transcranial motor evoked potentials (TcMEP) is a standard technique in modern neurosurgery. Because it enables real-time observation of functional integrity of the whole corticospinal tract with its supratentorial, brainstem and spinal segments, the implication of TcMEP monitoring goes beyond neurosurgical procedures. It includes orthopedic scoliosis surgery (Thirumala et al., 2016), carotid artery surgery (Malcharek et al., 2020) and lately endovascular intervention in stroke treatment (Sala et al., 2007, Shiban et al., 2016). Depending on the segment of the corticospinal tract (CST) to be monitored, TcMEP montage installation and interpretation may differ. This also applies to the different warning criteria that are implemented to detect an impeding risk of intraoperative CST injury. The variability in TcMEP installation and interpretation methods comes along with a set of pitfalls that hinder a 100% sensitivity and specificity in detection and prognostication of postoperative deficits. One of the most important aspects in eliciting TcMEP during surgery for supratentorial lesions is the required selectivity of the CST stimulation, a key requirement to avoid false negative results. This marks a difference with infratentorial, spine or scoliosis surgery, where TcMEP may be elicited by unselective stimulation in the depth of the subcortical white matter or at the level of the brainstem. The term “false negative results” refers to failure of TcMEP monitoring to detect an intraoperative injury of the CST, particularly due to a stimulation that bypasses the lesion and triggers the CST distally to the surgery site. The reason for that is either an unselective stimulation, a high current intensity, or a combination of both. In previous studies, our group addressed the performance of TcMEP during surgery for supratentorial lesions and developed a warning criterion that depends on evaluation of motor threshold (MT) defined as the lowest current intensity required to elicit motor action potentials from the monitored muscles bilaterally (Abboud et al., 2016, Abboud et al., 2018). In these works, we used the montage C3/C4 → Cz (according to the international 10–20 electroencephalography system) to elicit TcMEP. Although this electrode combination has been described as selective, a systematic comparison with other electrode configurations has not yet been performed. This study aims to distinguish the current intensity (MT) required for selective activation of the target CST from the intensity that leads to bilateral CST stimulation, reflecting activation of deeper white matter pathways extending to the brainstem. By doing so, we seek to determine the most selective electrode combination.

## 2 Materials and methods

### 2.1 Patients.

We prospectively enrolled patients who underwent surgery for a unilateral supratentorial lesion between January 2018 and January 2022, in whom transcranial electrical stimulation (TES) was performed to elicit TcMEP for monitoring of the integrity of the corticospinal tract. Inclusion criteria were patient age between 18 and 80 years, supratentorial gliomas, metastases, or meningiomas located in close proximity to the corticospinal tract (confirmed on a preoperative MRI), and absence of motor deficits on preoperative clinical examination. Exclusion criteria included a history of epileptic seizures and failure to elicit TcMEP bilaterally prior to surgery.

## 3 Anesthesia

Surgery was performed under general intravenous anesthesia. The same protocol was applied for all patients as detailed in previous publications (Abboud et al., 2021, Abboud et al., 2022). In summary, muscle relaxants were administered for intubation, anesthesia was induced using propofol, analgesia was applied by sufentanil for the intubation and continued with remifentanil. Invasive blood pressure monitoring was implemented in all patients to maintain stable systolic and mean arterial pressures. Monitoring began prior to the initiation of TES.

## 4 Installation of TES, TcMEP recording and determining the selectivity ratio

After induction of anesthesia, all patients were put in the supine position. Corkscrew-like electrodes were installed on the scalp according to the international 10–20 system at C1, C2, C3, C4 and Cz. Pairs of subdermal needle electrodes (Neurodart, Spes Medica) were inserted in the musculus abductor pollicis brevis (APB) bilaterally (100-ms epoch length, 1.5–853 Hz band-pass filter, 10,000x gain).

TES was conducted before skin incision and with a sufficient time interval of at least 45 min from the administration of muscle relaxants. Each of the following electrode-combinations was applied to elicit TcMEP: C1 ↔ C2, C3 ↔ C4 and C3/C4 → Cz. TcMEP were recorded from APB bilaterally.

The MT of contralateral APB, defined as the lowest current intensity that is necessary to achieve a muscle action potential of minimally 50 μV (Abboud et al., 2021), was determined for the contralateral APB. Afterwards, the current intensity was raised milliampere-wise until a muscle action potential could be provoked from the ipsilateral APB. This process was performed three times, i.e. once for each electrode combination (C1 ↔ C2, C3 ↔ C4 or C4/C3 → Cz).

For stimulation and recording, we used ISIS Xpert® and NeuroExplorer (V. 5.1) software (Inomed Medizintechnik GmbH, Emmendingen, Germany). TES applied a constant-current stimulation using a transcranial repetitive anodal 5-train- stimulation with an interstimulus interval of 4 ms, which was kept unchanged throughout the surgery. Pulse duration was 500 µs, and the train repetition rate was 1 Hz. Upper limit of stimulus intensity was 220 mA (mA).

### 4.1 Statistical analysis

For all tests, a p-value less than 0.05 was considered statistically significant. Statistical analysis and graphics were performed using IBM SPSS Statistics (v20, IBM Corp, Armonk, New York, USA), Microsoft Excel (2013, Microsoft Inc, Seattle, Washington, USA) and SigmaPlot (v12.5, Systat Software Inc, Erkrath, Germany).

Results of MT were presented as mean ± standard deviation and median [range]. The MT for contralateral and ipsilateral APB of a certain electrode combination were compared between both sides (affected hemisphere vs. healthy hemisphere) using a paired *t*-test. The averages of both sides were calculated and compared between different electrode-combinations using a paired *t*-test.

The selectivity ratio was defined as the ratio of the MT required to elicit an ipsilateral APB response to the MT required to elicit a contralateral APB response using the same electrode configuration. To facilitate interpretation, the selectivity ratio was expressed as a percentage according to the following formula: Selectivity ratio (%) = (Ipsilateral APB MT / Contralateral APB MT) × 100.

A higher selectivity ratio indicates a greater separation between the intensities needed for selective contralateral stimulation and those that cause bilateral activation, thereby reflecting higher selectivity of that electrode montage for isolated activation of the target CST.

The selectivity ratio (ipsilateral APB MT / contralateral APB MT) was calculated for each electrode-combination and compared between different combinations using paired *t*-test.

For analyzing the effect of age, weight, and height on stimulation values, we used linear regression (ordinary least squares, OLS).

## 5 Results

### 5.1 Patients:

**Fig. 1:**
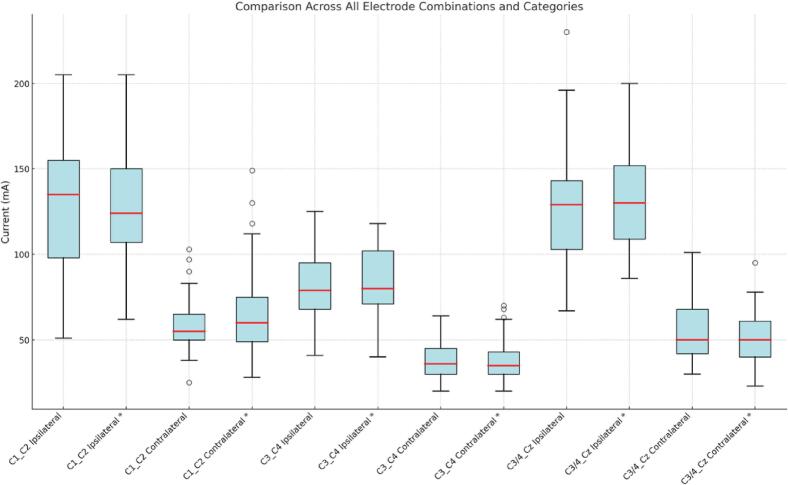
Boxplots comparing unaffected with affected (*) hemispheres across all electrode combinations and categories.

We included 56 patients with a mean age of 60 ± 17 years. Thirty-four patients (61%) were male. Among the cohort, thirty-nine patients (70%) were diagnosed with gliomas, ten with meningiomas (18%), and seven (12%) with metastases. The mean interval between muscle relaxant administration and TES application was 65 ± 7 min. Motor evoked potentials were successfully recorded in all patients for both ipsilateral and contralateral APB muscles. The mean and median MT of APB after ipsilateral and contralateral stimulation using different electrode combinations are presented in Table 1. There were no significant differences between affected and unaffected hemispheres across any electrode combination (Table 1 and Fig. 1).

**Table 1:** Paired T-Test results (with standard deviation and range) comparing stimulation parameter between the hemispheres (affected: with tumor vs. non-affected: without tumor).

Comparison	Mean 1 (SD)	Mean 2 (SD)	Median 1 (Range)	Median 2 (Range)	t-Statistic	p-Value
C1_C2 ipsilateral affected vs. non-affected	127.5 (37.2)	127.8 (33.3)	128.5 (51–205)	126 (60–205)	−0.055	0.957
C1_C2 contralateral affected vs. non-affected	61.4 (18.8)	69.1 (26.1)	59 (25–123)	66 (28–149)	−1.881	0.066
C3_4 ipsilateral affected vs. non-affected	89.7 (25.9)	93.5 (29.3)	85 (41–155)	90.5 (40–165)	−1.332	0.188
C3_4 contralateral affected vs. non-affected	44 (16.3)	42 (13.7)	40.(20–104)	38.5 (20–75)	0.966	0.338
C3/4_Cz ipsilateral affected vs. non-affected	131.1 (35.6)	135.85 (30.80)	130.0 (67–230)	133 (86–211)	−1.034	0.306
C3/4_Cz contralateral affected vs non-affected	57.21 (17.4)	53 (15.8)	53.5 (30–101)	50.5 (23–95)	1.367	0.178

## 6 Effect of sex, age, weight and height on stimulation values

The OLS analysis demonstrated no statistically significant differences in stimulation values between males and females across all electrode combinations. However, linear regression revealed a significant negative effect of age on contralateral MT for C1 ↔ C2 (p = 0.005), C3 ↔ C4 (p = 0.022), and C3/4 → Cz (p = 0.008), indicating a decline in MT with increasing age. For ipsilateral MT, a significant negative effect of age was observed only for C3 ↔ C4 (p = 0.001), suggesting a partial trend. Neither weight nor height showed significant associations with MT across any electrode combination.

## 7 Analysis of ipsilateral and contralateral measurements across different electrode combinations

Normality of the differences between ipsilateral and contralateral stimulation values was assessed using the Shapiro–Wilk test. As all paired differences were found to be normally distributed (p > 0.05), we used paired t-tests for comparisons. Accordingly, results are reported as mean ± standard deviation.

Mean MTs for ipsilateral and contralateral APB responses are summarized in Table 2.

**Table 2:** Mean ± standard deviation of motor thresholds and comparison p-values across electrode combinations.

Electrode combination	Side	Mean ± SD (mA)	Paired *t*-test (contralateral vs. ipsilateral)	p vs. C3–C4	p vs. C3/4–Cz
C1_C2	contralateral	64 ± 10		1.33E − 19	3.29E − 6
	ipsilateral	128 ± 32	1.42E − 15	2.73E − 10	0.953
C3_C4	contralateral	43 ± 8		—	2.03E − 22
	ipsilateral	92 ± 26	8.27E − 30	—	6.08E − 16
C3/4_Cz	contralateral	55 ± 9			—
	ipsilateral	132 ± 30	3.76E − 27		—

Ipsilateral thresholds were consistently and significantly higher than contralateral thresholds for each electrode combination (all p < 0.001). Among contralateral values, the highest threshold was observed for C1–C2, while the lowest was for C3–C4. For ipsilateral stimulation, C3/4–Cz and C1–C2 yielded the highest thresholds, with C3–C4 the lowest.

Pairwise comparisons between electrode combinations showed statistically significant differences in both ipsilateral and contralateral thresholds (Table 2). All contralateral comparisons were significant, while ipsilateral differences were significant between C1–C2 and C3–C4, and between C3–C4 and C3/4–Cz. No significant difference was found between C1–C2 and C3/4–Cz for ipsilateral stimulation.

**Fig. 2:**
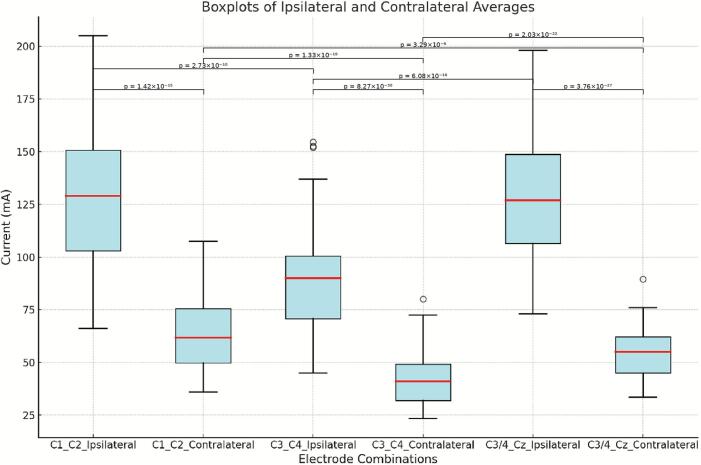
Boxplots showing ipsilateral and contralateral averages across all electrode combinations. Brackets indicate statistically significant differences.

Fig. 2 demonstrates MT averages of ipsilateral and contralateral APB using different electrode combinations.

## 8 Selectivity ratio

To assess the risk of simultaneous stimulation of bilateral CST, we calculated the selectivity ratios (ratio of ipsilateral to contralateral APB thresholds). This ratio represents the percentage increase in stimulation intensity required to evoke ipsilateral responses, presumably due to stimulation at the level of the brainstem. The mean ratios were: 214% ± 72% for C1 ↔ C2, 215% ± 33% for C3 ↔ C4, and 247% ± 40% for C3/4 → Cz.

**Fig. 3:**
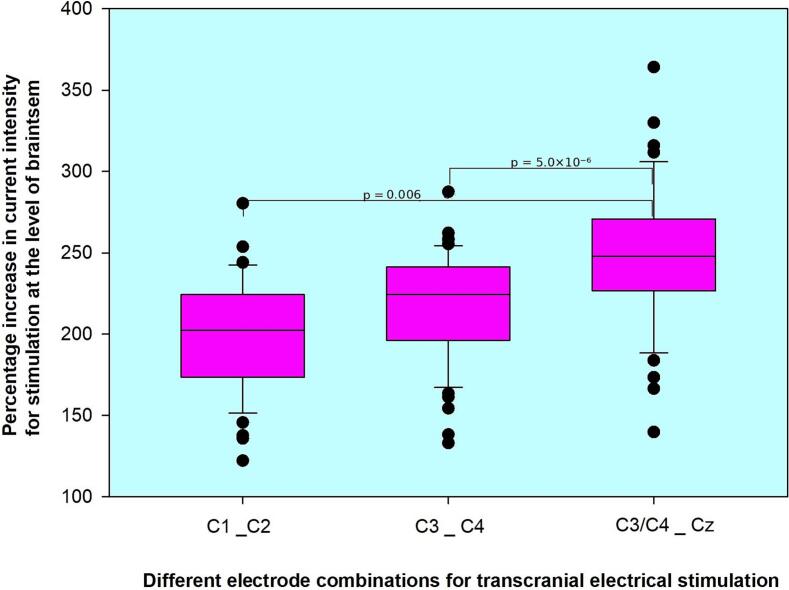
A line plot comparing the mean and median ratios of the motor threshold of ipsilateral APB to that of contralateral APB across the electrode combinations. The error bars represent the standard deviations for the mean values, while the square markers highlight the medians. Brackets indicate statistically significant differences.

Comparisons showed no significant difference between the ratios for C1 ↔ C2 and C3 ↔ C4 (p = 0.877), while significant differences were observed between the ratios for C1 ↔ C2 and C3/4 → Cz (p = 0.006), and between C3 ↔ C4 and C3/4 → Cz (p = 5.0 × 10^-6^). Fig. 3 compares calculated selectivity ratios across different electrode combinations.

## 9 Discussion

This study provides a comprehensive analysis of the selectivity of TES in stimulating the CST during IONM of supratentorial tumors. We investigated the most frequently used combinations in daily IONM practice including C1 ↔ C2, C3 ↔ C4 and C3/C4 → Cz. TcMEP were recorded from the APB, which represents the most reliable muscle in monitoring the integrity of corticospinal tract across numerous publications (Abboud et al., 2021, Kombos et al., 2009a, Kombos and Suss, 2009, Kombos et al., 2009b, Seidel et al., 2020, Szelényi et al., 2010, Szelényi et al., 2011). In each case included in this study, the MT of contralateral APB (to the tumor side) was established using each of the studied electrode combinations. Afterwards, MT of ipsilateral APB was established using each of the studied electrode combinations. Then the whole process was repeated on the unaffected (“healthy”) hemisphere.

The determined MT were similar in both hemispheres regardless of the used electrode combination as showed in Table 1 and Fig. 1, suggesting that the presence of a tumor itself may not affect the MT significantly. This allowed us to average the values of affected and unaffected hemispheres and compare between different electrode combinations. We found that the electrode combination C3 ↔ C4 elicits TcMEP with the lowest threshold levels. Yet associating low current intensities with higher selectivity may be misleading. The shortcomings of C3 ↔ C4 become clearer when comparing the current intensities needed to stimulate the ipsilateral APB, a stimulation that is deemed to occur at deeper white matter levels including the brainstem. Here, we observed significantly lower values for the montage C3 ↔ C4, whereas the settings C3/4 → Cz and C1 ↔ C2 needed higher intensities to elicit ipsilateral APB-TcMEP.

Interpreting absolute MT values only partially addresses the question of selectivity. Therefore, we computed the selectivity ratio, defined as the ratio of ipsilateral to contralateral MT, to capture the percentage increase required to elicit bilateral responses—reflecting deeper CST activation extending to the brainstem. The C3/4 → Cz montage exhibited the highest selectivity ratios, with statistically significant differences compared to both C1 ↔ C2 (p = 0.006) and C3 ↔ C4 (p = 5.0 × 10^−6^). This supports the hypothesis that C3/4 → Cz offers higher selectivity for isolated contralateral CST stimulation.

## 10 Significance of electrode combinations

Eliciting MEP via direct cortical electrical stimulation (DCS) has been suggested by many groups to be the gold standard (Kombos et al., 2009a, Kombos et al., 2000, Krieg et al., 2013, Krieg et al., 2012, Ritzl, 2012, Seidel et al., 2013). DCS indeed holds the anatomical advantage of performing stimulation directly at the cortical level, thereby needing much lower stimulation intensities. In comparison, eliciting TcMEP during supratentorial surgery requires higher stimulation intensities, which may bypass the lesion and either stimulate the CST distal to the lesion or even at the brainstem level. However, TcMEP remains reliable in situations where the motor cortex is not directly exposed, which applies to many supratentorial procedures. In contrast, DCS requires accurate placement of a strip electrode, which may be challenging or impossible when only a small craniotomy is performed. In addition, strip electrodes can dislocate during surgical manipulation, compromising continuous monitoring, and they are substantially more expensive than the screw electrodes used for TcMEP. For these practical reasons, TcMEP is often more feasible and broadly applicable technique for CST monitoring in routine supratentorial surgery.

In previous works, we established the TcMEP as a reliable method for monitoring the integrity of the corticospinal tract during supratentorial surgeries. We always used the combination C3/4 → Cz to evoke TcMEP and underscored its safety by comparing TcMEP bilaterally to incorporate changes resulting from brain shift (Abboud et al., 2017). In addition, a comparison between TES and DCS during surgery for supratentorial tumors is now being conducted in a prospective randomized multicenter study (the TRANSEKT trial)(Abboud et al., 2021).

**Fig. 4:**
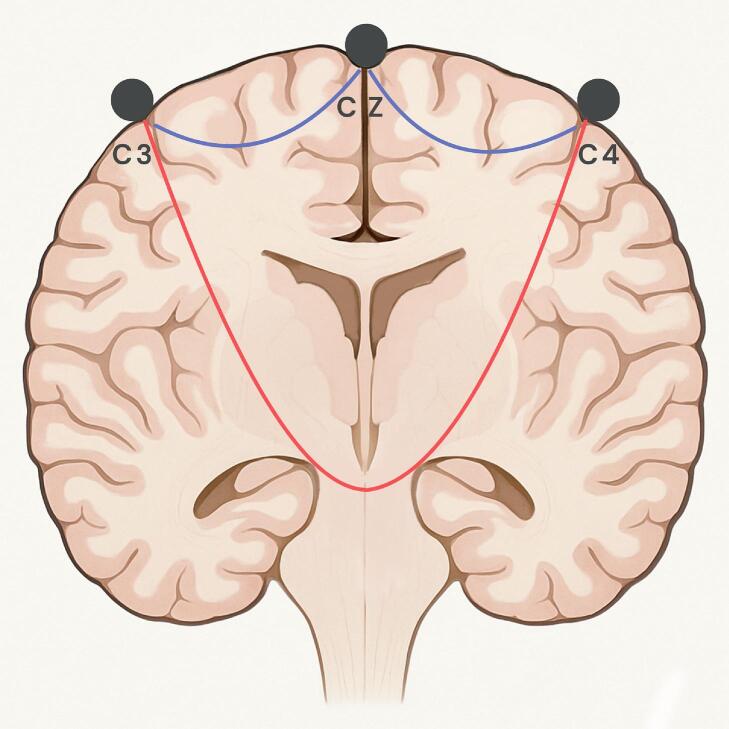
Illustration of specific electrode configurations for transcranial electrical stimulation. A schematic coronal view of the brain shows electrode positions according to the international 10–20 system: C3, C4, and Cz. Red arc represents potential current pathways for unselective bihemispherical transcranial stimulation (e.g., C3 ↔ C4), likely to engage brainstem structures. Blue arcs represent potential pathways for more selective, hemispheric stimulation (e.g., C3 → Cz or C4 → Cz), intended to target the CST unilaterally. (For interpretation of the references to colour in this figure legend, the reader is referred to the web version of this article.)

In the current study, the results provide robust proof for the selectivity of the C3/4 → Cz electrode combination, advocating its use whenever possible (Fig. 4). In addition, we demonstrated that eliciting bilateral TcMEP via deep white matter stimulation can already result from an increase in MT of 140% in case of C3/4 → Cz, 133% in C3 ↔ C4 and as low as 102% using the combination C1 ↔ C2. In the case of C1 ↔ C2 combination, the proximity of the two electrodes might result in an electrical shunt, i.e. a greater distribution of the electrical current across the scalp (Holdefer et al., 2006), which could explain the unselectivity of this combination in some of the observed cases.

Our results yielded some notably small p-values despite overlapping means and standard deviations. This is due to the use of paired t-tests, which assess within-subject differences. The consistency of these differences across patients leads to low variability and, consequently, highly significant p-values.

Recent work has explored alternative electrode montages designed to enhance the unilateral specificity of TcMEPs. Yamada et al. (2023) demonstrated that asymmetric bihemispheric electrode pairs— specifically C1(+)-C4(–) or C2(+)–C3(–)—can induce selective unilateral activation, however, without a direct comparison with conventional configuration (Yamada et al., 2023). Finite-element modelling work by Guo et al. provides a clear biophysical explanation for montage-dependent differences in TcMEP behavior. Although stimulation thresholds varied markedly across the tested bihemispheric configurations (LQP < M3–M4 < C1–C2), the simulations showed that the current density within the motor hand area at threshold was nearly identical across montages. This indicates that TcMEP activation occurs once a similar cortical current density is reached, independent of montage. The differences in threshold voltage were instead explained by variations in resistance along the current pathways, with lower resistance—and therefore lower thresholds—for montages with greater interelectrode separation (LQP, M3–M4) and higher resistance for more medial, closely spaced electrodes (C1–C2), as illustrated in the voltage-to-current–density relationships. Importantly, the modelling further demonstrated that local current–density topography was dominated by gyral geometry rather than scalp electrode location, resulting in similar cortical field distributions across montages. Together, these findings explain why certain electrode configurations appear more efficient clinically and emphasize that montage geometry—particularly electrode spacing—strongly influences TcMEP threshold behavior (Guo et al., 2023). Nevertheless, broader clinical validation across diverse surgical cohorts is needed before specific configurations can be universally recommended.

## 11 Effect of Age, Weight, and height

Linear regression analysis revealed a significant effect of age on contralateral and certain ipsilateral averages, suggesting an age-related increase in corticospinal tract excitability. This finding may be attributed to the age-associated decline in white matter volume and aligns with existing literature, which demonstrates that increasing age is associated with heightened cortical excitability—most likely due to reduced GABA-mediated intracortical inhibition (Heise et al., 2013, Opie and Semmler, 2014, Peinemann et al., 2001). In contrast, height and weight showed no significant influence, indicating these factors may have limited impact on stimulation thresholds. Recognizing the role of age may support the customization of intraoperative stimulation parameters to better suit individual patients.

## 12 Clinical Relevance

The results of this study have direct implications for clinical practice. The identification of optimal electrode combinations, such as C3/C4 → Cz, provides neurosurgeons with tools to enhance the reliability of IONM. The use of ipsilateral-to-contralateral ratios and distinct MT can improve the detection of unselective stimulation and reduce the likelihood of false-negative results. Furthermore, incorporating age as a predictive factor may improve monitoring accuracy in older patients.

## 13 Limitations

Although all montage configurations were tested before skin incision, intraoperative monitoring throughout the surgical procedure was performed using only one selected configuration. As a result, we could not compare the intraoperative diagnostic performance of the different montages in terms of sensitivity, specificity, or predictive values. This lack of comparative outcome data represents a major limitation of the present study and highlights the need for future studies designed to systematically evaluate the intraoperative performance of alternative TcMEP montages. We excluded patients with preoperative motor deficits or seizures. This likely resulted in a study population with relatively preserved CST integrity, even when lesions were close to the tract. Therefore, the thresholds observed here may not fully capture the behavior of TcMEPs in patients with preoperative weakness or substantial CST disruption, where altered excitability, higher thresholds, or reduced response reliability may occur. Caution is therefore warranted when generalizing our findings to such cases.

Although the mean interval between muscle relaxant administration and TES application was 65 ± 7 min, Train-of-Four monitoring was not performed. Therefore, a mild residual neuromuscular blockade cannot be fully excluded, representing a limitation of the study.

## 14 Conclusion

C3/C4 → Cz was identified as the most selective electrode combination for eliciting contralateral TcMEPs, showing the highest selectivity ratio. Notably, the risk of bilateral stimulation for this montage appears to begin at 140% of the contralateral muscle threshold. These findings highlight the importance of selectivity in TcMEP monitoring during supratentorial tumor surgery. Leveraging stimulation threshold ratios and incorporating age into monitoring protocols can enhance intraoperative.

## Funding

*“None of the authors have potential conflicts of interest to be disclosed.”*
